# Missed opportunities for HIV control: Gaps in HIV testing for partners of people living with HIV in Lima, Peru

**DOI:** 10.1371/journal.pone.0181412

**Published:** 2017-08-14

**Authors:** Ana L. Vasquez, Renato A. Errea, Daniel Hoces, Juan Echevarria, Elsa González-Lagos, Eduardo Gotuzzo

**Affiliations:** 1 Instituto de Medicina Tropical Alexander von Humboldt, Universidad Peruana Cayetano Heredia, Lima, Peru; 2 Facultad de Medicina, Universidad Peruana Cayetano Heredia, Lima, Peru; 3 Departmento de Enfermedades Infecciosas, Tropicales y Dermatológicas, Hospital Cayetano Heredia, Lima, Peru; University of Ottawa, CANADA

## Abstract

**Introduction:**

Based on the hypothesis that HIV programs struggle to deliver health services that harmonize necessities of treatment and prevention, we described the outcomes of routinely provided HIV testing to partners of people living with HIV (PLWH) through a secondary analysis of routine data collected at a public hospital in Lima, Peru.

**Methods:**

Among PLWH enrolled in the study center’s HIV program between 2005 and 2014, we identified index cases (IC): PLWH who reported a unique partner not previously enrolled. We grouped partners according to their HIV status as reported by IC and collected data on HIV testing, clinical characteristics and admissions. The main outcome was the frequency of HIV testing among partners with reported unknown/seronegative HIV status.

**Results:**

Out of 1586 PLWH who reported a unique partner at enrollment, 171 had a previously enrolled partner, leaving 1415 (89%) IC. HIV status of the partner was reported as unknown in 571 (40%), seronegative in 325 (23%) and seropositive in 519 (37%). Out of 896 partners in the unknown/seronegative group, 72 (8%) had HIV testing, 42/72 (58%) tested within three months of IC enrollment. Among the 49/72 (68%) who tested positive for HIV, 33 (67%) were enrolled in the HIV program. The proportion in WHO clinical stage IV was lower in enrolled partners compared to IC (37% vs 9%, p = 0.04). Non-tested partners (824) were likely reachable by the hospital, as 297/824 (36%) of their IC were admitted in the study center at least once, 51/243 (21%) female IC had received pregnancy care at the study center, and 401/692 (64%) of IC on antiretroviral therapy had achieved viral suppression, implying frequent visits to the hospital for pill pick-up.

**Conclusion:**

In this setting, HIV testing of partners of PLWH was suboptimal, illustrating missed opportunities for HIV control. Integration of HIV strategies in primarily clinical-oriented services is a challenging need.

## Introduction

The recommendation for initiating highly active antiretroviral therapy (HAART) to people living with HIV (PLWH) in serodiscordant couples has reinvigorated the notion that partners are a key target for HIV control [1,2]. Accordingly, the approach in which a diagnosis of HIV entails health services for partners of PLWH is expanding opportunities for HIV control.

Couple-based strategies, evaluated mainly under controlled research-settings in the United States [3], the sub-Saharan Africa and Latin America; have shown effectiveness for promoting safer sex behaviors [4], uptake of HIV counseling and testing [5], and adherence to medication [6, 7]. However, the implementation and routine delivery of services tailored to partners of PLWH by HIV programs are challenged by inadequate information systems, lack of training in couple-based modalities, stigma and gender-related barriers, and the sociocultural context of diverse local settings [3,8]. In addition, the legal framework that protects confidentiality of HIV status in some countries hampers the identification of partners [9–12].

Based on the working hypothesis that HIV programs struggle to deliver health services that harmonize responses for treatment and prevention needs, we retrospectively described the frequency and circumstances of HIV testing of partners of PLWH as routinely provided in a Peruvian public hospital during a ten-year period.

## Methods

### Study setting

Peru has a concentrated HIV epidemic with an estimated prevalence in general population close to 0.4% [13]. The Peruvian Ministry of Health regulates HIV care throughout the country and develops national HIV guidelines [14,16]. According to Peruvian law, HIV testing requires pretest counseling and signed informed consent except for pregnancy, blood and organ donation [17]. HIV testing is free of charge only if the individual has public health insurance [18]. Confirmatory tests for HIV, CD4 count, viral load tests and HAART are free of charge for all individuals who get enrolled to the HIV program of public hospitals [18].

The study center, a public hospital, is a national referral center for infectious and tropical diseases located in Lima. Through the HIV program (“The National Strategy for Control of Sexually Transmitted Diseases and HIV/AIDS”, formerly known as PROCETSS and currently named ESNITSS), the study center provides care, following the national HIV guidelines, to the largest number of PLWH in the country [18].

Briefly, the HIV program at the study center enrolls PLWH diagnosed there or at other health care facilities. During the study period, the HIV diagnosis strategy included a seropositive 3^rd^ or 4^th^ generation ELISA test performed at the center, followed by Western Blot or Indirect Immunofluorescence as confirmatory tests performed at the laboratory of the Peruvian Institute of Health. Rapid tests for HIV are only used at the Obstetrics and Gynecology (OBGYN) emergency room for women that arrive in labor and had not been under pregnancy care. Individuals are not aware of their ELISA test result unless they personally collect them. In case of seropositive ELISA results, trained nurses deliver the test result and provide post-test counseling. In parallel, a confirmatory test is requested. Given that the turnover time of confirmatory results is 2–4 weeks, individuals considered with high probability of having HIV by infectious diseases specialists can be enrolled in the HIV program with only a seropositive ELISA result.

At the time of enrollment in the HIV program, trained nurses ask PLWH “do you currently have partner(s)?”, where ‘partner(s)’ can mean any definition that the PLWH attributes. The nurse verbally encourages PLWH to disclose their HIV status to close relatives/friends and partner(s); the approach is individualized according to the nurse’s criteria. PLWH are also asked if they know their partner’s HIV status; whenever PLWH verbally report it as seronegative or unknown, HIV testing of the partner is verbally recommended to the PLWH. According to the national guidelines, the staff should encourage that HIV testing be repeated every 3 to 6 months in seronegative partners [17]. If the PLWH reports that the partner’s status is seropositive, no HIV testing is suggested; the nursing staff inquires if the partner is receiving care somewhere, but this is not registered and the routine records cannot verify such information. The baseline information of the PWLH and the information collected of the partner(s) (such as initials rather than the full name due to confidentiality issues) are filled out in structured forms and saved in the database of the HIV program.

Posteriorly, partners who voluntarily attend the HIV program for HIV testing follow the procedures above described. If diagnosed with HIV, partners will be invited to get enrolled in the HIV program and will thus be asked about their partner’s HIV status, as any other PLWH, because records of PLWH are not linked to that of a partner previously enrolled in the program.

### Study design and population

We conducted a secondary analysis of multiple routine data sources from the study center. Our main study outcome was the frequency of HIV testing among partners of PLWH whose HIV status was reported to be unknown or seronegative at the time of the PLWH’s enrollment to the HIV program.

We identified PLWH at least 18 years old enrolled in the HIV program at the study center between January 1, 2005 and December 31, 2014, and their partners. Follow-up was considered until March 31, 2015. We excluded PLWH who reported multiple partners as the registry of partners’ information required for our partner-PLWH matching strategy appeared to be less consistent in such cases.

### Study definitions

Index cases (IC) were defined as PLWH who reported a unique partner at the time of enrollment in the HIV program, provided that the reported partner had not been previously enrolled in the program.

HIV testing of the partner was the first ELISA test performed in the study center after enrollment of the IC. HIV testing was defined as timely if performed within three months of the IC enrollment, and delayed if done afterwards. Follow-up HIV testing was that ELISA test performed in the study center at least three months after the first ELISA above described.

Regarding clinical characteristics of interest, late testers were PLWH whose CD4 count was < 200 cell/mm^3^ within the first 100 days [19], and linkage to care was defined as a medical visit to the Infectious Diseases (ID) outpatient service within the first 30 days [20]. The hospital admission rate corresponded to the proportion of subjects with at least one admission per person-year during the study period. HAART eligibility at enrollment was based on the CD4 counts after diagnosis, according to the criteria in the national guidelines which changed over time: <200 cell/mm^3^ between 2005 and 2012 [14,16] and <350 cell/mm^3^ since 2012 [19]. Viral suppression was defined as a viral load <400 copies/mL at least six months after initiating HAART [14–16].

### Data sources and collection

We obtained the following information from four databases at the study center: (i) ELISA tests dates performed at the study center and their results; (ii) date of birth, sex, initials, number of partners, sexual orientation (the latter available only for those partners also enrolled in the HIV program), baseline CD4 count, viral load and HAART eligibility and use; (iii) dates of the visits at the ID outpatient service; (iv) dates and diagnosis of hospital admission(s). We corroborated with HIV program’s staff that the data collection strategies for our variables remained constant throughout the study period.

Routine registration systems at the study center did not track if partners of PLWH enrolled in the HIV program had been tested. Thus, in order to match IC and their corresponding partner, and to merge their data across the datasets, we created compound identifiers based on their year of birth, initials and sex. In the cases where both the IC and the partner were enrolled in the program, we verified that the IC was the first one enrolled, since neither the program nor the database made this distinction.

### Data measurements and analysis

As primary outcome, we reported the frequency of HIV testing among partners whose HIV status was reported by the IC as unknown or seronegative. As secondary outcomes, we assessed: (a) timely HIV testing of partners; and as a potential missed opportunity for HIV testing, (b) if the corresponding IC of non-tested partners had encounters at the study center that did not result in HIV testing of the partner. For the secondary outcome ‘b’, we considered the following as encounters: hospital admissions, pregnancy care at the study center and viral suppression (the latter as a proxy for hospital attendance since it implies monthly HAART pick-up at the HIV program office). Anticipating that the HIV status of a partner could be inaccurately reported, we examined how the partners enrolled in the HIV program reported the IC’s status, given that theoretically, it should be reported as seropositive. Finally, we did a paired comparison of clinical characteristics at the time of enrollment and during follow-up, between IC and the partners reported to have an unknown or seronegative HIV status, who subsequently had a positive HIV test and got enrolled in the HIV program.

The descriptive analysis was based, for categorical variables, on frequencies and percentages; for numeric variables with normal distribution on means and standard deviations, otherwise with medians and interquartile ranges. Prevalence differences were obtained using two-sample test for proportions with 95% confidence intervals (IC), and were considered to be statistically significant when p<0.05. The proportion of incomplete information was stated if missing values exceeded 5%. Data management and statistical analyses were done using STATA version 11 (license: Universidad Peruana Cayetano Heredia).

### Ethics statement

The study protocol was approved by the Institutional Review Boards (IRB) of Universidad Peruana Cayetano Heredia and Hospital Cayetano Heredia. The study was conducted according to CIOMS regulations for human subjects research, and in collaboration with the HIV program of the study center. Both IRB waived the need of written or verbal consent because the study was a secondary analysis of data collected as part of the HIV program routine activities.

## Results

### Study population

From January 2005 to December 2014, 3677 individuals ≥ 18 years old were enrolled in the HIV program at the study center: at the time of enrollment, 1586 (43%) reported a unique partner, 418 (11%) more than one partner and the remaining did not report any partner. A unique partner was more frequently reported in those who identified themselves as heterosexuals rather than non-heterosexuals (50.2%, 1134/2261 vs. 32.3% 443/1370, p < 0.01), the difference remained throughout the years.

**Fig 1:**
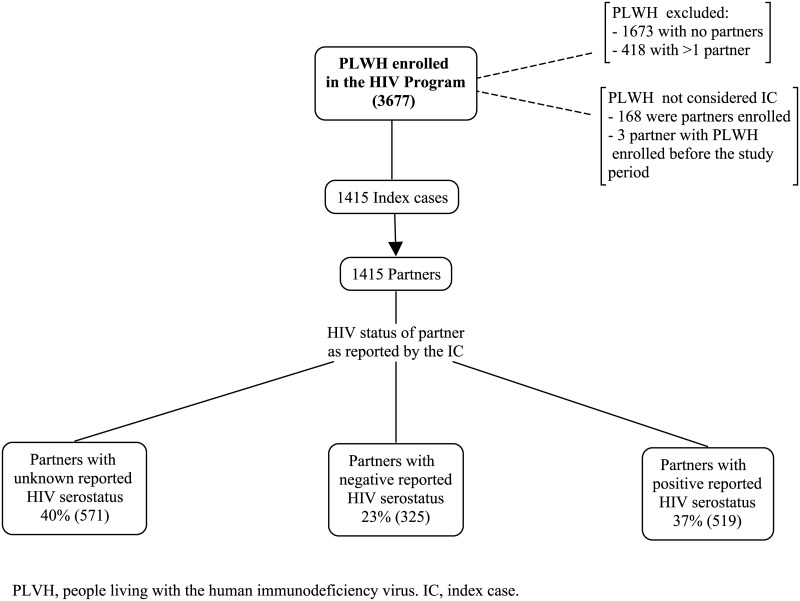
Identification and HIV status of partners.

Among the 1586 PLWH who reported a unique partner at the time of enrollment, 1415 were IC; 171 were not IC given that their partners, also PLWH, had been previously enrolled in the HIV program (Fig 1). According to our study definitions, 168 of these PLWH were partners enrolled in the HIV program; while in the remaining three, the PLWH that should had been the IC was enrolled before the start of our study period. Among the 168 partners enrolled in the HIV program, their report of the HIV status of their partner (IC) was mostly accurate, as 160 (94%) reported it as seropositive; yet, 6 (5%) reported it as unknown and 2 (1%) as seronegative.

### Characteristics of index cases and partners

The mean age (±SD) of the 1415 IC was 34.8 ±10.7 years; 907 (64%) were males. Most IC (999, 70%) identified themselves as heterosexuals. The mean age (±SD) of the 1415 partners was 34.1±10.8 years; 815/1378 (59%) were males. At the time of enrollment, the median length of the relationship with the reported partner was 4 years (interquartile range [IQR]: 1.6–9). Overall, 844 (60%) IC reported to know their partner’s HIV status, with no difference between heterosexuals (61%) and non-heterosexuals (59%). The HIV status of the partners was reported as unknown in 571 (40%), seronegative in 325 (23%) and seropositive in 519 (37%) (Fig 1).

### HIV testing of partners with unknown/seronegative HIV status

**Fig 2:**
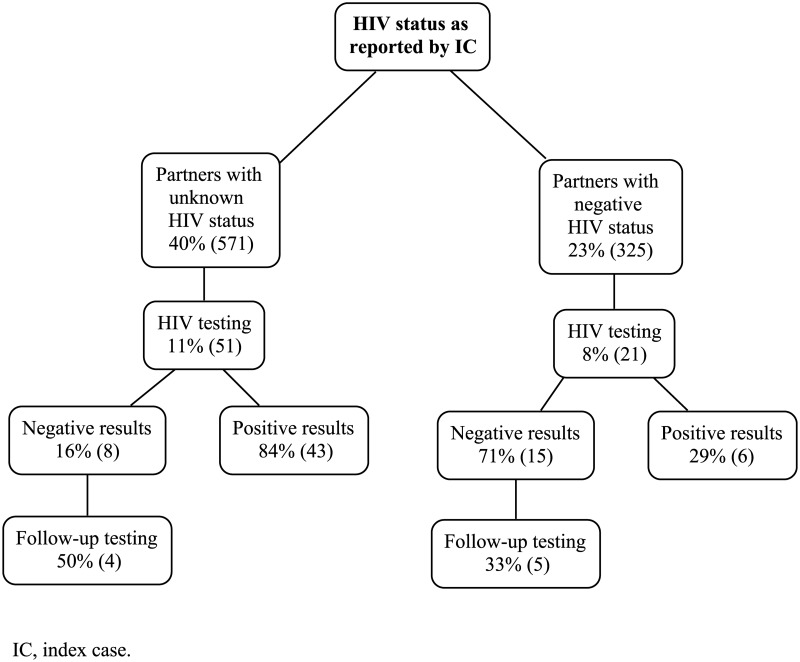
HIV testing of partners with unknown/seronegative HIV status.

**Fig 3:**
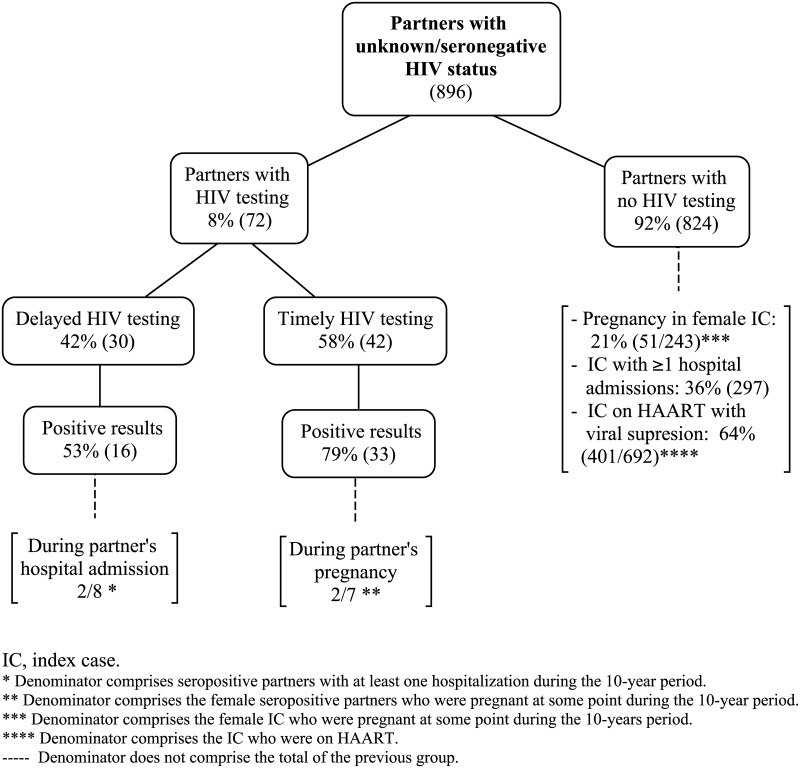
Circumstances and missed opportunities of HIV testing of partners.

**Fig 4:**
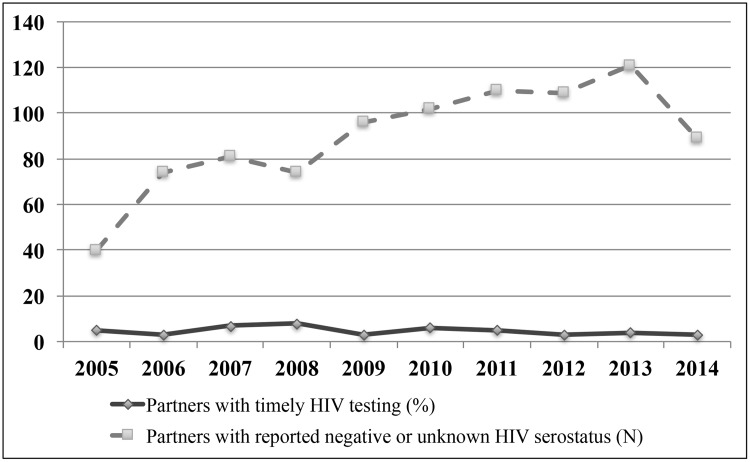
Partners with HIV timely testing throughout the 10-year period. No significant difference in the proportion of partners tested within three months of enrollment of IC and the total number of partners throughout the 10-year period (ANOVA, p = 0.28).

Until the end of the follow-up, 72/896 (8%) partners with reported unknown/seronegative HIV status underwent HIV testing at the study center after the date of enrollment of their IC (Fig 2). Among them, 42/72 (58%) had timely testing and 30/72 (42%) delayed testing (Fig 3), with no significant difference in the proportion of individuals timely tested and the total partners with unknown/seronegative HIV status throughout the ten-year period (ANOVA, p = 0.28, Fig 4).

Among the 42 partners timely tested, 33 (79%) were seropositive, of which 7 were women and 2 of them were tested for HIV during pregnancy. Among the 30 partners with delayed testing, 16 (53%) were seropositive, of which 8 had hospital admissions and 2 were tested for HIV during the admission (Fig 3).

According to the HIV testing results, 49/72 (68%) were diagnosed with HIV and 23/72 (32%) had seronegative results. Among the latter, 9 (39%) had follow-up testing and kept seronegative (Fig 2).

### Missed opportunities of HIV testing in partners

Among the 824 (92%) IC of non-tested partners, 297 (36%) had one or more hospital admissions, 51/243 (21%) female IC received pregnancy care at the study center, and 401/692 (64%) of IC on HAART had achieved viral suppression (Fig 3).

### Comparison of partners enrolled in the HIV program and their index cases

In the group of the 49/72 partners (initially reported with unknown/seronegative HIV status) diagnosed with HIV, 33 (67%) were enrolled in the HIV program. At the time of enrollment, the proportion in World Health Organization (WHO) clinical stage IV was higher among IC than in partners (37% vs. 9%, p = 0.04), without differences in the proportion of late testers and HAART eligibility between both groups. During follow-up, the hospital admission rate at the study center was significantly higher among IC compared to partners (11 vs. 3 per 100 person-years; p<0.01), without differences for linkage to care, viral suppression and mortality rate between both groups (Table 1).

**Table 1:** Comparison of clinical characteristics between partners enrolled in the HIV program and their index cases.

Clinical characteristics	Index cases (n = 33) N (%)	Enrolled partners* (n = 33) N (%)	Prevalence difference (CI)	P- value ^a^
**WHO clinical stage**				
I	6 (18)	4 (28)	-0.06 (-0.26, 0.14)	0.49
II	6 (18)	17 (52)	-0.34 (-0.55, -0.12)	<0.01
III	9 (27)	5 (15)	0.12 (-0.07, 0.31)	0.23
IV	12 (37)	3 (9)	0.22 (0.15, 0.42)	0.04
**Late tester**	14 (42)	10 (33)	0.09 (-0.14, 0.32)	0.45
**HAART eligibility**	20 (61)	15 (47)	0.14 (-0.09, 0.37)	0.25
**Linkage to care**	23 (70)	17 (52)	0.18 (-0.05, 0.41)	0.13
**Viral suppression**	16/30** (53)	13/23** (57)	0.04 (-0.27, 0.19)	0.74
**Hospital admission rate** ^b^	17/159 (11 x 100 person–years)	5/150 (3 x 100 person-years)	0.08 (0.02, 0.14)	<0.01
**Mortality**	1(3)	0(0)	0.03 (0.03, 0.09)	0.32

WHO, World Health Organization; HAART, highly active antiretroviral therapy

*Partners reported with unknown/seronegative HIV status at enrollment of IC, who subsequently tested positive and got enrolled in the HIV program

**Denominator represents those PLWH who had initiated HAART

^a^ *p* values were calculated using *x*^*2*^

^b^ Rate was calculated using person-years according to time of follow-up (from the date of enrollment to the HIV program to December 1, 2014)

## Discussion

Under routine conditions and across a ten-year period, the HIV testing of unique partners of PLWH enrolled in the HIV program at this particular public hospital was suboptimal, both in frequency and timing. This finding was established among reported partners whose HIV status was considered unknown or seronegative; the group that the local HIV program targets for HIV testing of partners. The limited identification of HIV-free partners and of undiagnosed HIV individuals imply missed opportunities for active HIV prevention [19,21] and for controlling morbidity rates of HIV among PLWH expected to be earlier diagnosed [22–24].

Health care of partners of PLWH starts with their identification. Expectedly, in this study, the absolute number of reported unique partners increased throughout the years as part of the overall increase in the number of individuals being diagnosed with HIV and enrolled in the program. However, the proportion of reported unique partners remained almost static throughout the years; however, which was also the case for the procedures that regulate HIV testing in our setting. At the same time, the proportion of reported unique partners remained higher for PLWH who identified themselves as heterosexuals rather than as non-heterosexuals. Among other reasons, this difference could be influenced by the different meanings of “partner” to non-heterosexual men [25], as well as the stigma associated with non-heterosexual relationships in the context of the current hetero-normative setting of Peru, making it less likely for non-heterosexual PLWH to report a partner to health workers [26,27]. At the same time, the reported knowledge of the HIV status of the partner was inferior (60%) in this hospital-based study compared to a population-based survey conducted in South Africa (77%) [28], which supports the need of disclosure services at the study center.

HIV testing of partners remained around 8% without any increase throughout the ten-year study period; and in this small group, it reached 39% for follow-up testing. Unaware of similar data in Latin America, our findings lag behind the 51% rate of HIV testing reported among partners of men who have sex with men (MSM) in US [29]. Our frequency of follow-up testing, though higher than the 24.5% reported by a study in sub-Saharan Africa [30], certainly points to gaps from the current global recommendation of once-a-year testing in high-risk individuals, such as partners of PLWH [31]. It should be noted that in Peru, HIV testing requires informed consent and pretest counseling, with strategies for self-testing still limited [32].

HIV testing of partners was suboptimal also in timing. First, the lapse between the enrollment of the IC and HIV testing of partners often exceeded three months. Second, whenever such testing was performed during admission and/or pregnancy care of partners, it did not necessarily respond to a specific partner-focused approach or prevention services delivered as part of the HIV care. Instead, it could be related to advanced disease that required hospital admission, or to routine counseling given to mothers as part of the prevention of mother-to-child transmission (PMTCT) program ongoing at the study center [33]. In addition, non-testing of some partners was observed in partners whose IC visited the study center after being enrolled in the HIV program. Such IC visits were related to pregnancy care, hospital admissions or routine care for HAART treatment, thus pointing to missed opportunities across health services offered at the study center. At a time when care of PLWH involves an increasing number of health services, strategic HIV prevention should be fostered beyond HIV programs involving key health services, particularly for the pool of individuals at high-risk, such as partners of PLWH [19,21,34,35].

In a context where the staff from the HIV program realizes the need and advocates for HIV prevention, we were intrigued by findings that non-testing occurred even among partners whose IC had repeated encounters at the study center. Accountability is paramount for programs’ performance [36]; yet, the accountability for the care of partners of PLWH was not clear within the scope of work of our local HIV program. The specific roles and responsibilities of health care personnel were not clearly defined, nor were systems that facilitated the proactive articulation of HIV programs with other hospital services (emergency room, OBGYN, surgery, etc.).

Disclosure of HIV status is a stressor for PLWH and can entail potential risks under certain circumstances. Therefore, it could be the case that health care staff postponed or adapted the emphasis on such recommendations; or that PLWH decided against doing so [30,37]. Indeed, problems with disclosure in our setting [38], could explain why in some cases the HIV status of IC was sometimes inaccurately reported as negative or unknown by their partners. Moreover, given that late entry into care of IC prevailed, recommendations on disclosure and HIV testing of partners could become sidetracked and/or limited to initial encounters [30,37].

The specific and immediate disease-related needs of advanced HIV can be particularly pressing both for PLWH and health care services, competing in priority with prevention services [39] unless the latter are fully in place. Primarily clinical-oriented services are a rich niche for expanding prevention activities through PLWH [40]. Yet, effective evidence-based interventions for care of PLWH together with their partners, such as couple counseling and testing [2], assisted disclosure [41], and services for partner notification of HIV diagnosis [42] were not locally implemented. In fact, it is quite illustrative that the local routine registration systems did not record if partners of PLWH enrolled in the HIV program had been tested or were receiving care elsewhere [37].

Other circumstances could further challenge the care of partners willing to be tested for HIV. Except for pregnant women, HIV tests without out-of-pocket expenses had limited availability at the study center; even though procedures to waive such costs and obtain a public health insurance were progressively implemented, administrative completion of such waiver could demand one full workday. Thus, facilitating free of cost tests for partners of PLWH who are receiving care at the study center’s HIV program may increase the uptake of HIV testing in partners. Similarly, broader access to HIV rapid testing for the general population should also be considered within the HIV program activities as they have shown to be a fast and cost-effective HIV screening tool in hospital-based settings [43].

Across the HIV continuum of care, undiagnosed HIV individuals constitute a major challenge with approximately half of the PLWH population remaining undiagnosed [44,45]; this is the case in many settings, such as in Latin American countries, where figures reach 30% in Argentina, 60% in Venezuela [8], and in Peru, low detection rates of HIV-infected people have been reported as the main local gap in the continuum of care [13]. Nevertheless, detection of infected individuals is just the first step in the cascade of care. A recent study at our center that examined retention in care during a six-year period determined that after one year of enrollment in the HIV program, less than 60% of PLWH were retained in care [46]. In our study, although the majority of the partners diagnosed with HIV were enrolled in the HIV program, it did not happen for all cases. In addition, the better outcomes seen among the small group of partners enrolled in the HIV program support the reported clinical benefits of early entry into care of PLWH [22–24]. Thus, suggesting the positive effects of partner-identification and linkage strategies.

Among the main limitations of this study, we categorized partners based on their HIV status as reported by the IC at enrollment in the HIV program; however, such report may not be completely accurate, was not updated in time, and did not establish if disclosure to partners had occurred. Additionally, given that our definitions align with the HIV program approach for partner prevention strategies, partners tested prior to the IC enrollment were not explored; although admittedly, they may yield interesting information regarding HIV testing of partners in other situations. Further studies should be performed to understand the circumstances previously described, as well as for detecting those partners tested for HIV or receiving care elsewhere, as this information was not available in our databases. Furthermore, we excluded PLWH with multiple partners because the routine procedures appeared to be less consistent in such cases. We hypothesize that this may not have caused much change in the results due to the low percentage (11%) of PLWH reporting more than one partner in our cohort, but this topic deserves further studies. Finally, in spite of <5% missing data on the database and a rigorous matching strategy, we expect that not a 100% of the couples were identified.

Despite these limitations, we think that our findings consistently illustrate a series of missed opportunities in the care of PLWH and their partners that ultimately hinder the approach of treatment as prevention and the HIV continuum of care. Beyond specific interventions of undeniable necessity, we need to improve the wellbeing of PLWH, their families and the community, and should work toward providing health care to PLWH and their partners outside the exclusive domain of HIV programs.
